# Prevalence and Impact of Obesity in Patients With Diabetes and Myocardial Infarction: A SWEDEHEART Registry Report

**DOI:** 10.1016/j.jacadv.2025.101644

**Published:** 2025-03-13

**Authors:** Viveca Ritsinger, Bo Lagerqvist, Anna Norhammar

**Affiliations:** a Cardiology Unit, Department of Medicine K2, Karolinska Institutet, Stockholm, Sweden; b Department of Research and Development, Region Kronoberg, Växjö, Sweden; c Department of Medical Sciences, Cardiology, and Uppsala Clinical Research Center, Uppsala University, Uppsala, Sweden; d Capio S:t Görans Hospital, Stockholm, Sweden

**Keywords:** acute myocardial infarction, coronary angiography, diabetes, obesity, prognosis

Diabetes and obesity are common and important cardiovascular (CV) risk factors and obesity is one major contributor to diabetes development. The combination of diabetes and obesity increases the mortality and CV risk dramatically.^1^ Lifestyle interventions are important, difficult to sustain, and with limited evidence on CV-preventive effects.^2^ Therefore, the novel finding of CV protective effects with weight-reducing glucagon-like peptide receptor agonists (GLP-1 RAs) in obesity is encouraging and will revolutionize the present dismal scenario. However, since this drug-class also is important for cardioprotection in diabetes, there are emerging challenges for health care societies in how to prioritize among those with diabetes or obesity, since GLP-1 RA at present comes with high costs, shortage of supply, and inequality of access. This study aimed to explore the prevalence of overweight and obesity in a real-life cohort of patients with diabetes and acute myocardial infarction (AMI) and how obesity affects outcome.

We included all patients (n = 25,907) with diabetes with a non-ST-segment elevation myocardial infarction (NSTEMI) or ST-segment elevation myocardial infarction (STEMI) in 2010-2021 that underwent coronary angiography in Sweden and were included in the SWEDEHEART registry. Information on body mass index (BMI) was collected at the time of angiography and grouped into: low BMI (<18.5 kg/m^2^), normal BMI (18.5-24.9 kg/m^2^), overweight (25.0-29.9 kg/m^2^), obesity grade 1 (30.0-34.9 kg/m^2^), obesity grade 2 (35.0-39.9 kg/m^2^), and obesity grade 3 (≥40.0 kg/m^2^). Diabetes diagnosis was obtained from the National Patient Registry (International Classification of Diseases-10 codes E10-E14) and SWEDEHEART. Patients were followed for MI, stroke, heart failure, and renal failure until December 31, 2021, and all-cause death until March 31, 2022, via national registries. The Swedish Ethical Review Authority approved the study (Dnr 2012/60-31/2, 2017/432-32, 2020-04252).

Baseline characteristics and outcomes were compared across the BMI classes (presented as mean [IQR] for continuous variables and numbers [%] for categorical variables). Mortality in the first 30 days was excluded from analysis (regarded as the index hospitalization). Cox proportional hazards analyses were performed adjusted for age, sex, smoking, year of inclusion, indication, angiographic findings, previous diagnosis of MI, heart failure, cancer, hypertension, hyperlipidemia, renal failure, stroke, and peripheral artery disease. Normal BMI served as a reference. A 2-sided *P* value of <0.05 was considered statistically significant. Analyses were conducted using the R-statistical program (version 4.2.2) (R Core Team).

In all, 66% (n = 17,186/25,907) were men, 37% (n = 9,587/25,907) suffered a STEMI. Normal BMI was present in 21.7% (n = 5,610/25,907), overweight in 41.1% (n = 10,653/25,907), obesity grade 1 in 25.0% (n = 6,478/25,907), obesity grade 2 in 8.5% (n = 2,212/25,907), obesity grade 3 in 3.1% (n = 805/25,907), and underweight in 0.6% (n = 149/25,907). Patients with obesity grade 3 vs normal BMI were younger (64 vs 72 years), more often women (45% [n = 363/805] vs 36% [n = 2,026/5,610]), had more frequent heart failure (11% [n = 90/805] vs 7% [n = 410/5,610]), and hypertension (81% [n = 648/798] vs 69% [3,803/5,504]), while a higher proportion of those with normal BMI had suffered a previous MI and stroke (all with *P* < 0.001). Patients with underweight were the oldest (75 years), more often women (64%; n = 95/149) and smokers (27%; n = 40/149), and with a more severe comorbidity pattern than the other BMI groups. GLP-1 RA was used in 14% (n = 109/805) of patients with obesity grade 3 compared to 2% (n = 110/5,610) in those with normal BMI. When only analyzing patients included in 2021, 30% (n = 24/80) of patients with obesity grade 3 were on GLP-1 RA compared to 8% (n = 35/448) of those with normal BMI. Corresponding figures for sodium glucose-lowering transport 2 receptor inhibitors in 2021 were 45% (n = 36/80) in patients with obesity grade 3 compared to 34% (n = 153/448) in those with normal BMI.

Figure 1A illustrates the generally high event rate in patients with diabetes post-MI during a mean follow-up time of 2,175 (IQR: 1,112-3,245) days. Apart from those with underweight, the absolute 1-year event rate for major adverse cardiovascular event (MACE; first of all-cause death, MI, stroke, heart failure) and mortality was highest in patients with normal BMI (34.8%; n = 1,800/5,172 and 15.7%; n = 811/5,165 respectively). In comparison, the absolute 1-year event rate for hospitalization for heart failure (16.2%; n = 119/735) and renal failure (9.4%; n = 69/734) was highest in those with obesity grade 3 (despite being younger). Associated risks of adverse outcomes across BMI groups are depicted in Figure 1B. Compared to normal weight, the adjusted hazard risk of future MACE was increased in obesity grade 2; HR: 1.10 (95% CI: 1.02-1.18) and obesity grade 3; HR: 1.32 (95% CI: 1.19-1.46). The associated risk of all-cause death was increased in patients with obesity grade 3 (HR: 1.27 [95% CI: 1.11-1.46]) while there was no statistical difference in risk of future MI and stroke between the BMI groups. Regarding heart failure and renal failure, the associated risk was increased for all grades of obesity compared to normal BMI and highest in obesity grade 3 (HR: 1.81 [95% CI: 1.58-2.08] and HR: 1.62 [95% CI: 1.37-1.91]).

**Figure 1:**
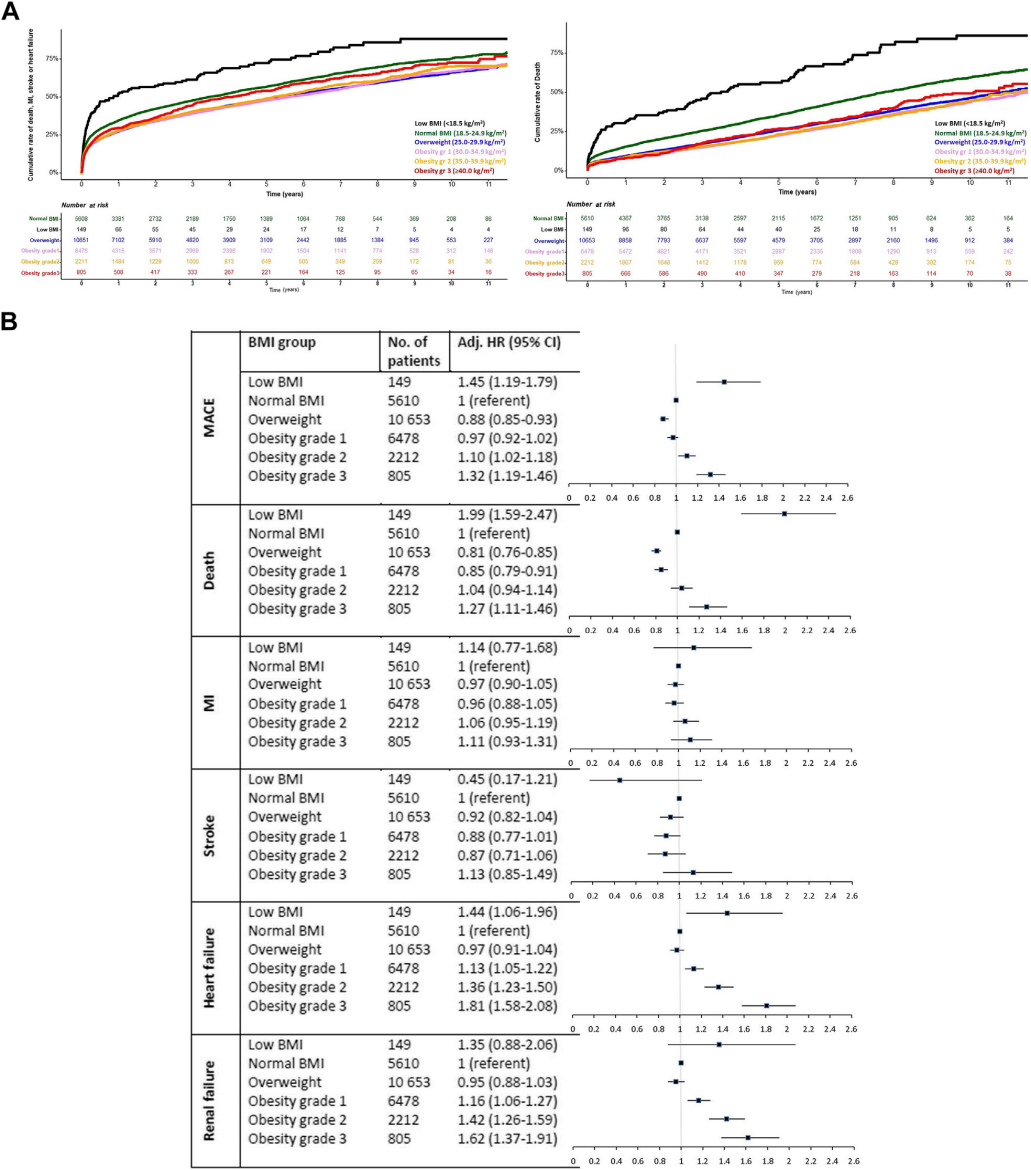
Cumulative Event Rates of MACE and Mortality (Survival Curves) and Adjusted Cardiovascular Risks (Forest Plot) by BMI in Patients With Diabetes and Myocardial Infarction in the SWEDEHEART-Registry (A) Time to all-cause MACE (all-cause death, myocardial infarction, stroke, or heart failure; left) and death (right) by classes of BMI (black = low BMI, green = normal BMI, blue = overweight, pink = obesity grade 1, orange = obesity grade 2, red = obesity grade 3). (B) Adjusted-associated HR (95% CI) for MACE, all-cause death, myocardial infarction, stroke, heart failure, and renal failure by BMI group during a mean follow-up of 6 years (reference = normal BMI). MI = myocardial infarction.

In this large, unselected cohort of patients with diabetes and AMI, there are 3 major findings. First, obesity is common among AMI patients with diabetes affecting around one-third, and only one-fifth have normal weight. In the general population in Sweden, obesity is lower, present in around 16%. Secondly, a minority were on cardioprotective GLP-1 RA, and with very low use in those with obesity grade 3. Third, obesity grade 3, present in rather few, was associated with the highest risks of future MACE and all-cause death while *all* grades of obesity were associated with increased risk of heart failure and renal failure. International guidelines recommend that all with type 2 diabetes and CV disease (CVD) should be on novel cardioprotective glucose-lowering drugs. Our data show that obesity indeed is common in MI patients with diabetes and that having all 3 diseases; MI, diabetes, and obesity; is a morbid triad, associated with a higher risk for disabling complications especially heart failure and renal failure. Our data support that novel cardioprotective glucose-lowering agents really should be of high priority in this group at high risk for novel events. In Sweden, sodium glucose-lowering transport 2 receptor inhibitors are increasingly used in diabetes after an AMI while GLP-1 RAs are of low use^3^ and as we show here, of very low use even if obesity grade 3 is present. Given the worldwide problem with high costs and in particular the shortage of GLP-1 RA, we believe patients with diabetes, CVD, and obesity should be prioritized, and absolutely those with grade 3 obesity.

A limitation with the present study is the lack of information on hemoglobin A1c, type of diabetes, duration, glucose-lowering treatment, and distribution of adipose tissue (including waist, liver, and epicardium). Furthermore, the high event rates in normal BMI were not elaborated on but are most likely due to the higher age in those with normal weight compared to those with obesity.

In conclusion, this study demonstrates a high prevalence of obesity in patients with diabetes and CVD and highlights the associated increased risk of a misfortunate outcome. These data support that in patients with diabetes and AMI, obesity grade 2 and 3 should absolutely be prioritized for GLP-1 RA, and thereafter those with normal BMI/overweight.

## Funding support and author disclosures

This work was supported by the Swedish Heart-Lung Foundation and the Department of Research and Development Region Kronoberg. Dr Ritsinger has received honoraria on expert group participation from AstraZeneca, Novo Nordisk, and Boehringer Ingelheim. Dr Norhammar has received honoraria on expert group participation from AstraZeneca, Eli Lilly and Company, Novo Nordisk, and Boehringer Ingelheim. Dr Lagerqvist has reported that he has no relationships relevant to the contents of this paper to disclose.
